# Case Report: Carcinosarcoma of the common bile duct: a rare case report with literature review

**DOI:** 10.3389/fonc.2025.1642467

**Published:** 2025-08-18

**Authors:** Jia-qi Lou, Zhi-Wei Hu, Kun Liu, Ru-La A, Hao-Yu Fang

**Affiliations:** ^1^ Department of Surgery, Inner Mongolia Medical University, Hohhot, China; ^2^ Department of Hepatobiliary Surgery, Inner Mongolia People’s Hospital, Hohhot, China; ^3^ Department of Hepatobiliary Surgery, Beijing Friendship Hospital, Capital Medical University, Beijing, China

**Keywords:** carcinosarcoma, common bile duct, pancreaticoduodenectomy, sarcomatoid differentiation, prognosis

## Abstract

Carcinosarcoma of the common bile duct is an exceedingly rare and highly aggressive malignancy with uncertain therapeutic outcomes. We present a case of a 70-year-old Asian female presenting with progressive jaundice. Contrast-enhanced computed tomography (CT) identified a tumor in the common bile duct. The patient underwent successful radical pancreaticoduodenectomy, and postoperative histopathological analysis confirmed carcinosarcoma with predominant sarcomatoid differentiation. This report underscores the diagnostic challenges and therapeutic considerations for this rare entity, emphasizing the critical role of pathological confirmation.

## 1 Introduction

Carcinosarcoma is a rare biphasic malignancy comprising both epithelial-derived carcinoma and mesenchymal sarcomatoid components. While commonly reported in the breast and female reproductive system, its occurrence in the biliary tract is exceptionally rare. Clinical manifestations, imaging findings, and serological markers of biliary carcinosarcoma lack specificity, necessitating histopathological confirmation for definitive diagnosis. Due to its aggressive nature and propensity for metastasis, prognosis remains poor, with limited evidence guiding optimal therapeutic strategies. This report details a rare case of carcinosarcoma of the common bile duct and synthesizes current literature to highlight diagnostic and therapeutic challenges.

## 2 Case report

A 70-year-old woman presented with a two-week history of progressive jaundice, right upper abdominal distension, fatigue, dark urine, and clay-colored stools. Her medical history was unremarkable for hepatitis, liver disease, or chronic comorbidities. Physical examination revealed scleral icterus and a weakly positive Murphy’s sign.

Liver function tests (**Table 1**) and Tumor markers (**Table 2**) showed elevated levels:

**Table 1:** Elevated liver function test results.

Test	Value	Unit
Aspartate aminotransferase (AST)	474.00	U/L
Alanine aminotransferase (ALT)	687.00	U/L
Gamma-glutamyl transferase (GGT)	758.00	U/L
Total bilirubin	186.38	μmol/L
Direct bilirubin	169.08	μmol/L

**Table 2:** Elevated tumor marker levels.

Test	Value	Unit
Carbohydrate antigen 19-9 (CA19-9)	547.900	U/mL
Carbohydrate antigen 242 (CA242)	87.780	U/mL
Tissue polypeptide-specific antigen (TPS)	534.510	U/L

**Figure 1:**
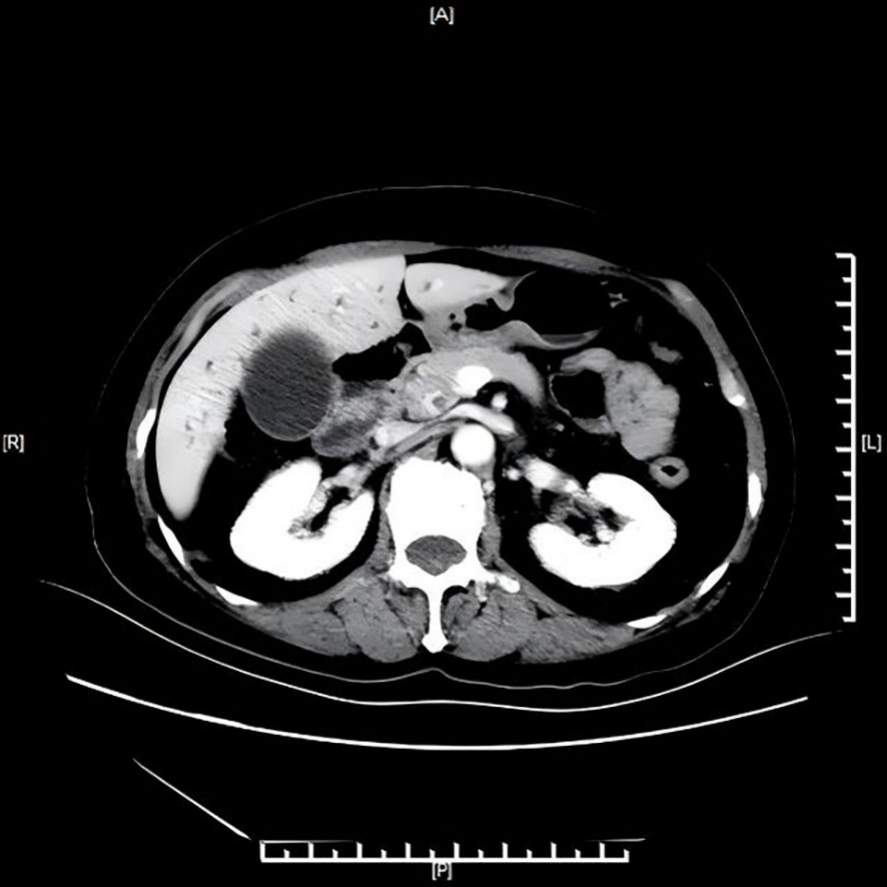
Contrast-enhanced CT showing distal biliary obstruction with ductal dilation.

Abdominal color Doppler ultrasound revealed hypoechoic nodules in the ampulla of the duodenum behind the pancreatic head at the end of the common bile duct, along with intrahepatic and extrahepatic bile duct dilatation, hepatic cysts, and an enlarged gallbladder. Contrast-enhanced CT (**Figure 1**) showed uneven thickening of the wall at the pancreatic segment and junction of the common bile duct, suggesting malignant expansion of the lower biliary tract, with possible involvement of the pancreatic head. Hepatic S7 cyst was also noted.

**Figure 2:**
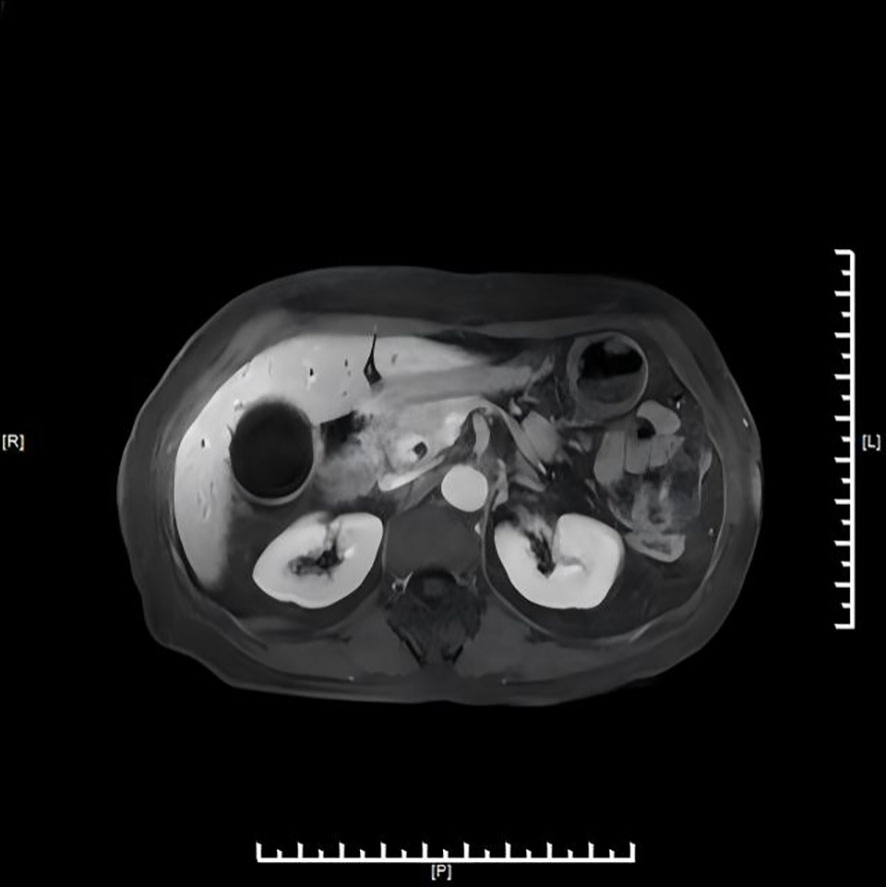
MRI/MRCP demonstrating low cystic duct insertion and irregular thickening at the cystic duct–common hepatic duct junction.

Hepatobiliary contrast-enhanced Magnetic Resonance Imaging (MRI) with diffusion-weighted imaging (DWI) and magnetic resonance cholangiopancreatography (MRCP) (**Figure 2**) revealed irregular thickening at the confluence of the gallbladder duct and hepatic duct, with the thickened portion measuring approximately 8 mm. DWI showed a slightly higher signal, and the enhanced scan demonstrated significant enhancement. Hepatic S7 cyst was again noted.

**Figure 3:**
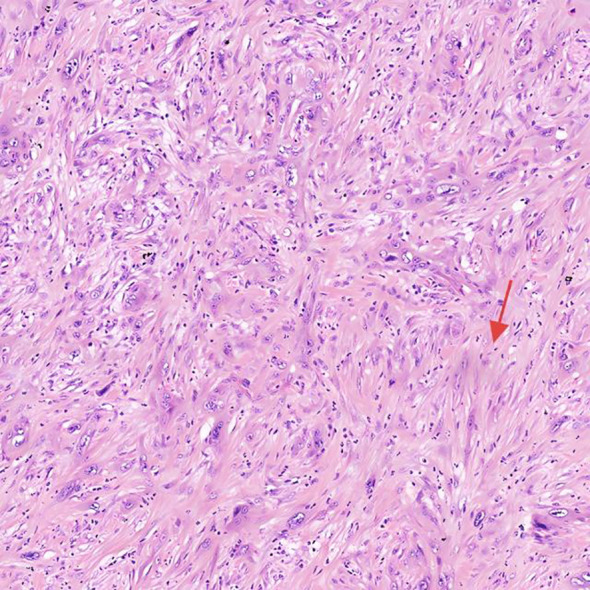
Histopathological and immunohistochemical features H&E of the tumor, highlighting sarcomatoid differentiation and high proliferative activity.

**Figure 4:**
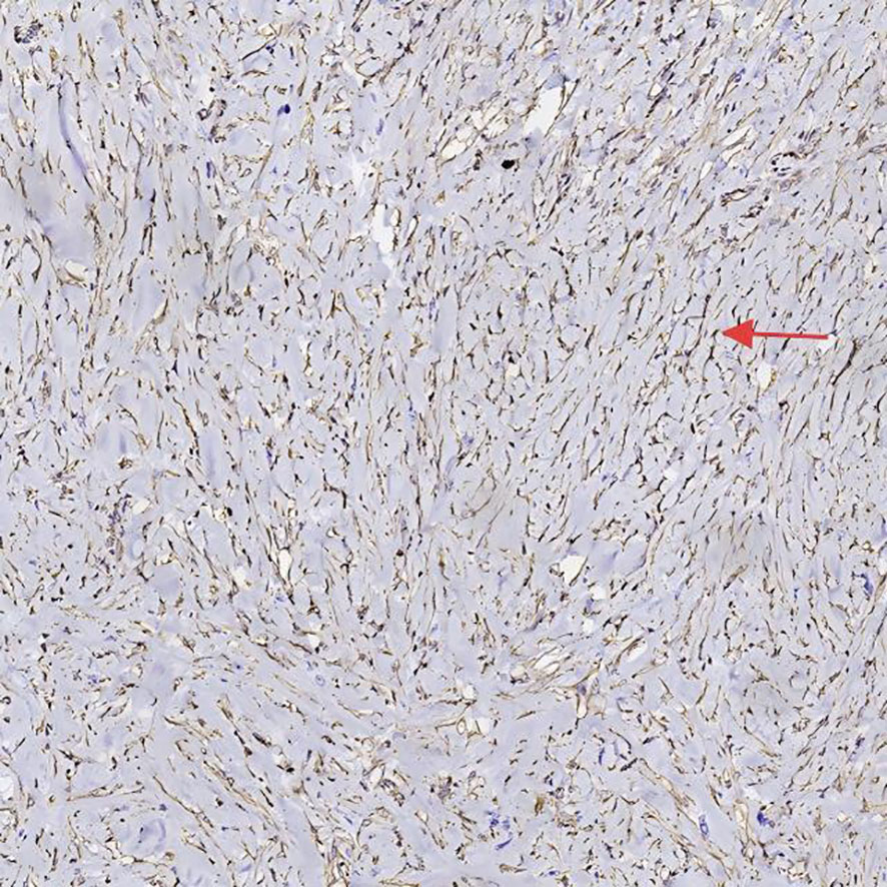
Histopathological and immunohistochemical features VIM of the tumor, highlighting sarcomatoid differentiation and high proliferative activity.

After preoperative evaluation and multidisciplinary discussion, the patient underwent radical pancreaticoduodenectomy with hepatic cyst incision and drainage on November 4, 2024. The surgery was successful. Postoperative pathological examination (**Figures 3**, **4**) revealed poorly differentiated adenocarcinoma of the common bile duct with sarcomatoid and squamous differentiation, predominantly sarcomatoid components. The tumor measured approximately 2.5 cm x 1.0 cm x (0.3-1.0 cm), invading the entire wall of the common bile duct and surrounding adipose tissue, with micro-invasion of the pancreas and suspected intravascular cancer emboli. No metastasis was found in the duodenum, pancreas, or lymph nodes. Immunohistochemistry results were as follows: CK (+), CK7 (+), CK19 (+), CK20 (partially weak +), MUC-2 (-), CDX2 (partially weak +), VIM (-), SMA (-), S-100 (-), Desmin (-), CK5/6 (focal +), P63 (-), BRG-1 (+), Glypican-3 (-), P53 (individual cells +), and ki-67 (+60-70%).

The patient developed a grade C pancreatic fistula postoperatively and was discharged 40 days after surgery. Follow-up contrast-enhanced CT more than five months post-surgery showed no tumor recurrence.

## 3 Discussion

### 3.1 Introduction

The concept of carcinosarcoma was first described by the German pathologist Virchow in 1864 (1).This rare and highly aggressive malignancy is characterized by the coexistence of epithelial-derived carcinoma and mesenchymal-derived sarcomatoid tissue, with the sarcomatoid component often predominating. Car cinosarcomas can arise in various organs, including the breast, pancreas, lung, uterus, ovary, esophagus, stomach, and kidney (2–9). According to Sasamoto et al., breast cancer (approximately 39.8% of cases) and uterine cancer (24.9%) represent the most common sites of carcinosarcoma (10). Recent epidemiological studies indicate a rising incidence of carcinosarcoma, particularly in the female reproductive and respiratory systems. Notably, carcinosarcoma of the digestive system is associated with the poorest prognosis, with a median survival period of approximately six months (11).

### 3.2 Etiology and pathogenesis

The origin of carcinosarcoma remains a subject of ongoing debate, with three primary hypotheses proposed in the literature. The first, proposed by Wiebren van den Berg et al., suggests that carcinosarcoma arises from phenotypic diversity within monoclonal tumors. This hypothesis is supported by molecular studies of mucinous cystic tumors of the pancreas, which demonstrated nearly identical genetic alterations between sarcomatous and epithelial components in two out of three cases. In the third case, allele loss and retention were identical in five of six chromosomal loci (12). The second hypothesis, termed the “metaplasia theory,” posits that sarcomatoid components result from the metaplastic transformation of epithelioid cancer tissues (13). The third hypothesis, the “collision theory,” proposes that carcinosarcoma arises from the independent development and subsequent fusion of carcinoma and sarcoma tissues, which originate from distinct cell lineages (14).

### 3.3 Clinical presentation and diagnostic challenges

The clinical manifestations of cholangiocarcinoma sarcoma are nonspecific and closely resemble those of conventional cholangiocarcinoma. Okabayashi et al. reported that the mean age of patients with extrahepatic cholangiocarcinoma sarcoma is approximately 68 years, with obstructive jaundice being the most common presenting symptom (15). Imaging studies typically reveal polypoid masses within the bile duct, a finding also observed in carcinosarcomas of other hollow organs, such as the esophagus and bladder (16). Common imaging features include bile duct dilation and biliary obstruction, as demonstrated by cholangiography and computed tomography (CT). However, these findings are not pathognomonic for carcinosarcoma and may also be observed in conditions such as polypoid cholangiocarcinoma, gallbladder cancer with bile duct involvement, pseudotumors, or metastatic disease. Consequently, definitive diagnosis relies on histopathological examination.

### 3.4 Therapeutic approaches and prognosis

Carcinosarcoma is characterized by its high malignant potential, aggressive local invasion, and propensity for metastasis. Lee et al. have suggested that the sarcomatoid component is primarily responsible for the tumor’s invasiveness, as it exhibits a greater tendency to metastasize to lymph nodes and distant organs (17). Radical surgical resection remains the cornerstone of treatment. For intrahepatic cholangiocarcinosarcoma, surgical options include partial liver resection (e.g., hemihepatectomy, lobectomy, or segmentectomy) combined with lymph node dissection. For extrahepatic cholangiocarcinosarcoma, pancreaticoduodenectomy or tumor resection with choledochojejunostomy is performed, depending on the tumor’s anatomical location (18). The choice of surgical approach is guided by the tumor’s location, extent, and progression, with operative time, blood loss, and transfusion requirements varying accordingly. In the case presented by the authors, the tumor was located in the common bile duct, and radical pancreaticoduodenectomy was performed following multidisciplinary discussion.

### 3.5 Survival outcomes and adjuvant therapy

Okabayashi et al. conducted a comprehensive review of clinicopathological data from 131 patients who underwent surgical resection for hepatobiliary carcinosarcoma between 1970 and 2012. The overall survival rates at 1, 3, and 5 years post-surgery were 44.0%, 29.3%, and 27.0%, respectively. Among these, 24 patients had extrahepatic cholangiocarcinoma sarcoma, with 1-year, 3-year, and 5-year survival rates of 43.3%, 28.9%, and 28.9%, respectively (15). Several case studies highlight the challenges associated with treating choledochal carcinosarcoma. For instance, Suguru Sasamoto et al. reported a patient who developed liver metastasis nine months post-pancreaticoduodenectomy and succumbed to the disease 26 months after surgery (10). Similarly, Kadono et al. described a patient who underwent pylorus-preserving pancreaticoduodenectomy but experienced local recurrence and died two years postoperatively (19). Tanaka et al. reported a patient who died ten months after radical pancreaticoduodenectomy (20). In contrast, Zhang Shuisheng et al. documented a patient who received adjuvant chemotherapy (cisplatin and gemcitabine) two months post-surgery and remained disease-free at three years of follow-up (21). Emerging evidence suggests that chemotherapy regimens incorporating docetaxel and gemcitabine may improve response rates and survival outcomes in sarcomatoid carcinoma (22). These findings underscore the potential benefit of combining radical surgery with tailored adjuvant chemotherapy to enhance patient prognosis.

## 4 Conclusion

This case highlights the diagnostic complexity and aggressive nature of carcinosarcoma of the common bile duct. Preoperative differentiation from other malignancies is challenging, underscoring the indispensability of histopathology. Radical surgery remains the primary therapeutic option, but multimodal approaches incorporating chemotherapy may improve outcomes. Future research should focus on standardized adjuvant protocols and molecular profiling to refine management strategies.

## Data Availability

The original contributions presented in the study are included in the article/**Supplementary Material**. Further inquiries can be directed to the corresponding author/s.
