# Supplementary figures and images for: Case Report: Carcinosarcoma of the common bile duct: a rare case report with literature review
Source: Front Oncol. 2025 Aug 18;15:1642467. doi: 10.3389/fonc.2025.1642467 (PMC12399393; doi:10.3389/fonc.2025.1642467)

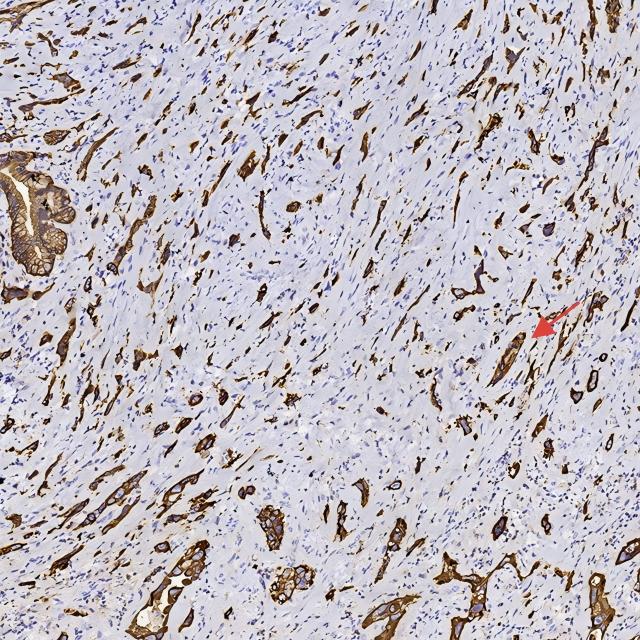

Supplement: Supplementary file 1 [file Image1.jpeg]

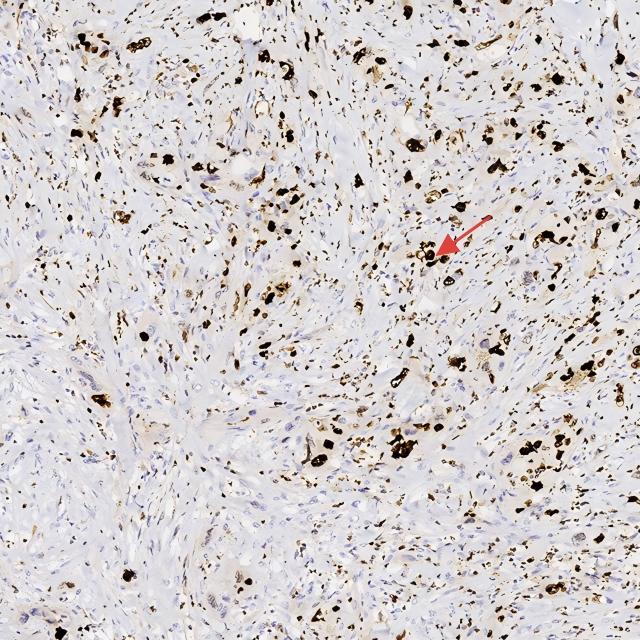

Supplement: Supplementary file 2 [file Image2.jpeg]
